# Delivery of platelet *TPM3* mRNA into breast cancer cells via microvesicles enhances metastasis

**DOI:** 10.1002/2211-5463.12759

**Published:** 2019-11-21

**Authors:** Bing Yao, Shuang Qu, Ruifeng Hu, Wen Gao, Shidai Jin, Junyi Ju, Quan Zhao

**Affiliations:** ^1^ The State Key Laboratory of Pharmaceutical Biotechnology School of Life Sciences Nanjing University China; ^2^ Department of Oncology The First Affiliated Hospital of Nanjing Medical University China

**Keywords:** biomarker, breast cancer, platelet, RNA, *TPM3*

## Abstract

Platelets are implicated in the pathophysiology of breast and other cancers through their role in exchanging biomolecules with tumor cells in the tumor microenvironment. Such exchange results in tumor‐educated platelets with altered RNA expression profiles. Multiple lines of evidence indicate that platelet RNA profiles may be suitable as diagnostic biomarkers for cancer‐related biological processes. In this study, we characterized the gene expression signatures of platelets in breast cancer (BC) by high‐throughput sequencing and quantitative real‐time RT‐PCR. Our results indicate that the expression of *TPM3* (*tropomyosin 3*) mRNA is significantly elevated in platelets from patients with BC compared with age‐matched healthy control subjects. Furthermore, up‐regulation of *TPM3* mRNA in platelets was found to be significantly correlated with metastasis in patients with BC. Finally, we report that platelet *TPM3* mRNA is delivered into BC cells through microvesicles and leads to enhanced migrative phenotype of BC cells. In summary, our findings suggest that the transfer of platelet *TPM3* mRNA into cancer cells via microvesicles promotes cancer cell migration, and thus platelet‐derived *TPM3* mRNA may be a suitable biomarker for early diagnosis of metastatic BC.

## Abstract 2

Platelets are implicated in the pathophysiology of breast and other cancers through their role in exchanging biomolecules with tumor cells in the tumor microenvironment. In this study, we found that platelet *TPM3* (*tropomyosin 3*) mRNA can be delivered into cancer cells via microvesicles to promote cancer cell migration. Platelet‐derived *TPM3* mRNA may be a suitable biomarker for early diagnosis of metastatic breast cancer.

## Graphical abstract

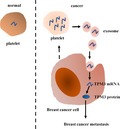

**Abbreviations**
BC: breast cancer
EMT: epithelial‐to‐mesenchymal transition
NSCLC: non‐small‐cell lung cancer
qRT‐PCR: quantitative real‐time RT‐PCR
ROC: receiver operating characteristic curve
siRNA: small interfering RNA
TEP: tumor‐educated platelet
TPM3: tropomyosin 3
WGCNA: weighted correlation network analysis

Breast cancer (BC) is the second leading cause of cancer deaths after lung cancer in women 1. The 5‐year relative survival rate for women diagnosed with localized BC is 99%; however, it decreased to 27% for the patients with metastasis 1. Therefore, there is an urgent need to develop novel, especially noninvasive surrogate biomarkers for early screening of metastatic BC.

Platelets are involved in multiple steps of cancer 2. A large number of studies previously established that platelets could be educated by tumor cells when they were exposed to the tumor and its microenvironment by exchanging biomolecules, including nucleotides and proteins 2, 3. During this process, intraplatelet signaling of tumor cells was triggered, thereby resulting in alteration of RNA profiles, splicing of platelet pre‐mRNAs and enhanced secretion of cytokines 2. Furthermore, platelets have been found to be capable of sequestering RNAs and proteins released by cancer cells 2, 3. These platelets, in turn, promote tumor progress, including tumor cell survival, immune escape and metastasis 2, 3. The transcriptome and molecular content of platelets appear to be dynamically affected by tumors and could accurately reflect the cancer progression 4, 5. Therefore, platelets have been recognized as perfect resources of actual blood‐based liquid biopsy for cancer diagnosis and management, especially the RNAs in platelets 4. Platelets contain many RNA species, including mRNAs, small noncoding RNAs and circular RNAs 5, 6. Platelet RNAs have been confirmed to be dynamically affected by tumor conditions and possibly used as biomarkers for cancer diagnosis, prognosis, prediction or monitoring 5, 6. For example, Calverley *et al*. 7 profiled by microarray analysis the platelet mRNA of healthy volunteers and patients with metastatic lung cancer without any treatment. They found 200 RNAs were altered between the healthy volunteers and the patients with lung cancer, and platelet RNA could serve as a potential biomarker for metastatic lung cancer 7. Nilsson *et al*. 8 found that platelets of patients with glioblastoma could sequester tumor‐specific epidermal growth factor receptor variant III (EGFRvIII) RNA from tumor cells by microvesicle, and the EGFRvIII RNA transcripts in platelets were detected with a sensitivity of 80% and a specificity of 96%. Moreover, they also found a panel of RNA genes in the platelets that could distinguish between patients with glioblastoma and healthy control subjects 8. Later, the same group confirmed the uptake of tumor‐derived transcripts, including translocated EML4‐ALK, in patients with non‐small‐cell lung cancer (NSCLC) 9. Best *et al*. 10 performed RNA sequencing of platelets from 55 healthy controls and 228 patients with cancer with different tumor types (including NSCLC, colorectal cancer, pancreatic cancer, glioblastoma, BC and hepatobiliary carcinomas). Compared with platelet samples of healthy donors, 1453 out of 5003 mRNAs were increased, whereas 793 out of 5003 mRNAs were decreased in TEPs 10. Then, using the differentially expressed mRNA profiles of patients with cancer and healthy donors, they developed a predictive algorithm with high accuracy rate (96%) in separating healthy individuals from patients with cancer 10. Moreover, they also found that the RNA profiles of platelets could be used to classify the tumor type (accuracy rate 71%) and molecular mutational subtype (accuracy rate 85–95%) 10. Later, the same group confirmed that the algorithm enabled the diagnosis of late‐stage NSCLC with an accuracy rate of 89% and locally advanced stage I–III NSCLC with an accuracy rate of 81% 11. Hence these studies suggested that platelet RNA appears to be a potential biomarker for tumor diagnosis, prognostic, prediction or monitoring.

In this study, we characterized the gene expression signatures of platelets in BC by high‐throughput sequencing and quantitative real‐time RT‐PCR (qRT‐PCR) assays. These results suggested that the expression of *TPM3* (*tropomyosin 3*) mRNA was significantly elevated in the platelets from patients with BC compared with the age‐matched healthy controls. Moreover, the expression level of *TPM3* mRNA was also up‐regulated in the platelets from patients with metastasis compared with the patients without metastasis. In addition, we confirmed *TPM3* mRNA in the platelets was delivered into BC cells through microvesicles and led to an enhanced migrative phenotype of BC cells.

## Materials and methods

### Patient characteristics

Blood samples from 549 patients with BC without any treatment and 154 age‐matched healthy volunteers were included in this study. All patients with BC were diagnosed at the First Affiliated Hospital of Nanjing Medical University. The clinicopathological data of all patients with BC were collected, and the characteristics of the patients and healthy control subjects enrolled in the training and validation sets are given in Table 1. This study was approved by the Ethics Committee of the First Affiliated Hospital of Nanjing Medical University, and written informed consent was obtained from all patients. All experiments were performed in accordance with relevant guidelines and regulations. Protocols were designed and performed according to the principles of the Declaration of Helsinki.

**Table 1:** Patient characteristics and clinical features.

Characteristics	Normal Subjects (*n* = 154)	Patients with BC (*n* = 549)
Age (y)	54.5 ± 18.4	58.1 ± 13.3
Sex (*n*)		
Female	154	549
ER status (*n*)		
Positive		424
Negative		125
PR status (*n*)		
Positive		369
Negative		180
Histological type (*n*)		
Ductal carcinoma		393
Lobular carcinoma		102
Mucinous carcinoma		8
Metaplastic carcinoma		5
Medullary carcinoma		3
Mixed histology		38
Stage (*n*)		
I		95
II		318
III		125
IV		11
T (*n*)		
1		145
2		327
3		56
4		21
N (*n*)		
0		266
1		182
2		71
3		30
M (*n*)		
0		539
1		10

### RNA profiles of platelets for BC

The RNA profile of platelets from BC by Illumina HiSeq 2500 (Shallowater, TX, USA) was downloaded from the Gene Expression Omnibus database (GSE68086). The dataset, including 23 platelet samples from patients with BC and 45 platelet samples from healthy volunteers, was first performed to normalization by limma package, and the differentially expressed genes were subsequently analyzed by ebayes. Then, we performed weighted correlation network analysis (WGCNA) to identify the coexpression network and the hub genes, which could be the potential biomarkers for the BC diagnosis.

### Platelet isolation and RNA extraction

Whole blood was collected into heparinized tubes and centrifuged at 300 ***g*** for 30 min to separate platelet‐rich plasma. Platelets were isolated from platelet‐rich plasma at 3000 ***g*** for 30 min. Then, the platelets were washed with 1 mL of 1× PBS in the presence of prostaglandin E1 (50 ng·mL^−1^) at 3000 ***g*** for 30 min three times. Subsequently, total RNAs were isolated from platelets by the RNeasy Mini Kit (Qiagen, Hilden, Germany) according to the protocols.

### qRT‐PCR

qRT‐PCR was performed with a 7300 Sequence Detection System (Applied Biosystems, Foster City, CA, USA) using the TaqMan® Reverse‐Transcription Kit and the TaqMan® Fast Advanced Master Mix (Applied Biosystems) according to the manufacturer’s instructions. All reactions, including no‐template controls, were run in triplicate. The primers used in the study were given in Table 2. The primer for *TPM3* pre‐mRNA was listed as follows: forward 5′‐TCCTCTTACGGGGTGCTCTT‐3′; reverse 5′‐GTTCCTGCCTTCCAGGTCAT‐3′.

**Table 2:** Selection criteria of mRNAs from the screening phase. GEO, Gene Expression Omnibus.

Gene symbol	Platelet (GEO)	
	Fold change	*P*‐value
*HIST1H2BC*	3.271638	9.93E−9
*HIST1H2BK*	2.605881	3.72E−7
*STXBP2*	2.10066	2.35E−5
*SSX2IP*	2.174059	2.07E−5
*HIST1H2AC*	2.630354	9.54E−7
*TPM3*	2.096225	3.14E−5
*YIF1B*	2.688851	4.93E−8
*MAGED2*	2.615131	4.08E−7

### Microvesicle isolation and incubation with MDA231

To isolate platelet microvesicles, we extracted platelets from patients with BC and healthy volunteers and resuspended them in Tyrode’s buffer (150 mmol·L^−1^ NaCl, 5 mmol·L^−1^ HEPES, 0.55 mmol·L^−1^ NaH_2_PO_4_, 7 mmol·L^−1^ NaHCO_3_, 2.7 mmol·L^−1^ KCl, 0.5 mmol·L^−1^ MgCl_2_ and 5.6 mmol·L^−1^ glucose). The supernatant was centrifuged again to prepare a platelet‐free releasate after 48 h at 3000 ***g*** for 30 min, which was used for platelet microvesicle isolation. Platelet microvesicles were harvested by centrifugation at 10 000 ***g*** for 1 h at 4 °C in a TL‐100 ultracentrifuge (Beckman Coulter, Pasadena, CA, USA) and were either resuspended in HEPES‐Tyrode buffer for cell coincubations, extracted for RNAs by TRIzol (Invitrogen, Carlsbad, CA, USA) or extracted for proteins by radioimmunoprecipitation assay lysis buffer. The protein concentration was calculated by the bicinchoninic acid protein assay kit (Thermo Scientific, Rockford, IL, USA). For incubation of platelet microvesicles with MDA231, MDA231 cells were seeded on 12‐well dishes, and platelet microvesicles (500 µg total proteins) isolated from patients with BC or healthy volunteers were added into each well. After incubation for 24 h, MDA231 cells were collected for qRT‐PCR and the quantitative protein assay.

### Western blotting

Proteins of cells and microvesicles were extracted by radioimmunoprecipitation assay lysis buffer, and western blot analysis was performed as previously reported 12. In brief, an equal amount of extracted protein was separated on a 10% SDS/PAGE, followed by being transferred to a polyvinylidene difluoride membrane under the condition of 300 mA for 1 h (Tannon, Shanghai, China). After blocking with 5% nonfat milk in 1× TBST (TBS, 0.1% Tween 20) buffer for 1 h, the membrane was incubated with primary antibodies prepared with 5% nonfat milk in 1× TBST for 1 h at room temperature. After washing with 1× TBST three times, 20 min each time, secondary antibody incubation at a dilution of 1 : 10 000 was performed for 1 h at room temperature. The membrane was washed with 1× TBST four times and detected on a gel imaging system using enzyme chemiluminescence western blotting substrate (Thermo Fisher Scientific, Waltham, MA, USA), and band density was analyzed with imagej software (National Institutes of Health, Baltimore, MD, USA). The antibodies were purchased as follows: TPM3 (3D5AH3AB4; Abcam, Cambridge, UK) and GAPDH (M171‐3; MBL International, Beijing, China).

### Cell invasion assays

The *in vitro* cell invasion of MDA231 cells was assessed using the transwell assay according to the manufacturer’s protocol 12. In brief, 5 × 10^5^ cells incubated with 500 μg of platelet microvesicles were seeded into the upper chamber of the transwell apparatus (Corning Costar, Waltham, MA, USA), which was precoated with 50 μL of a Matrigel solution in serum‐free medium, and medium supplemented with 15% FBS was added to the bottom chamber. After 24 h, the cells on the upper surface that did not pass through the 8‐μm pore‐size polycarbonate filter were removed using a moistened cotton swab; the cells migrating to the lower membrane surface were fixed in 100% methanol for 20 min, stained with 0.4% crystal violet for 20 min and counted under a microscope (Nikon, Tokyo, Japan) at 100× magnification.

### Statistical analysis

Statistical analysis was performed with spss 16.0 software (IBM, Almonk, NY, USA). Student’s *t*‐test or two‐sided χ^2^ test was used to compare the differences in other variables among the groups. A *P*‐value < 0.05 was considered to be statistically significant. Receiver operating characteristic curve (ROC) analysis was performed to estimate the diagnostic value of platelet mRNAs.

## Results

### Identification of differentially expressed genes

**Figure 1:**
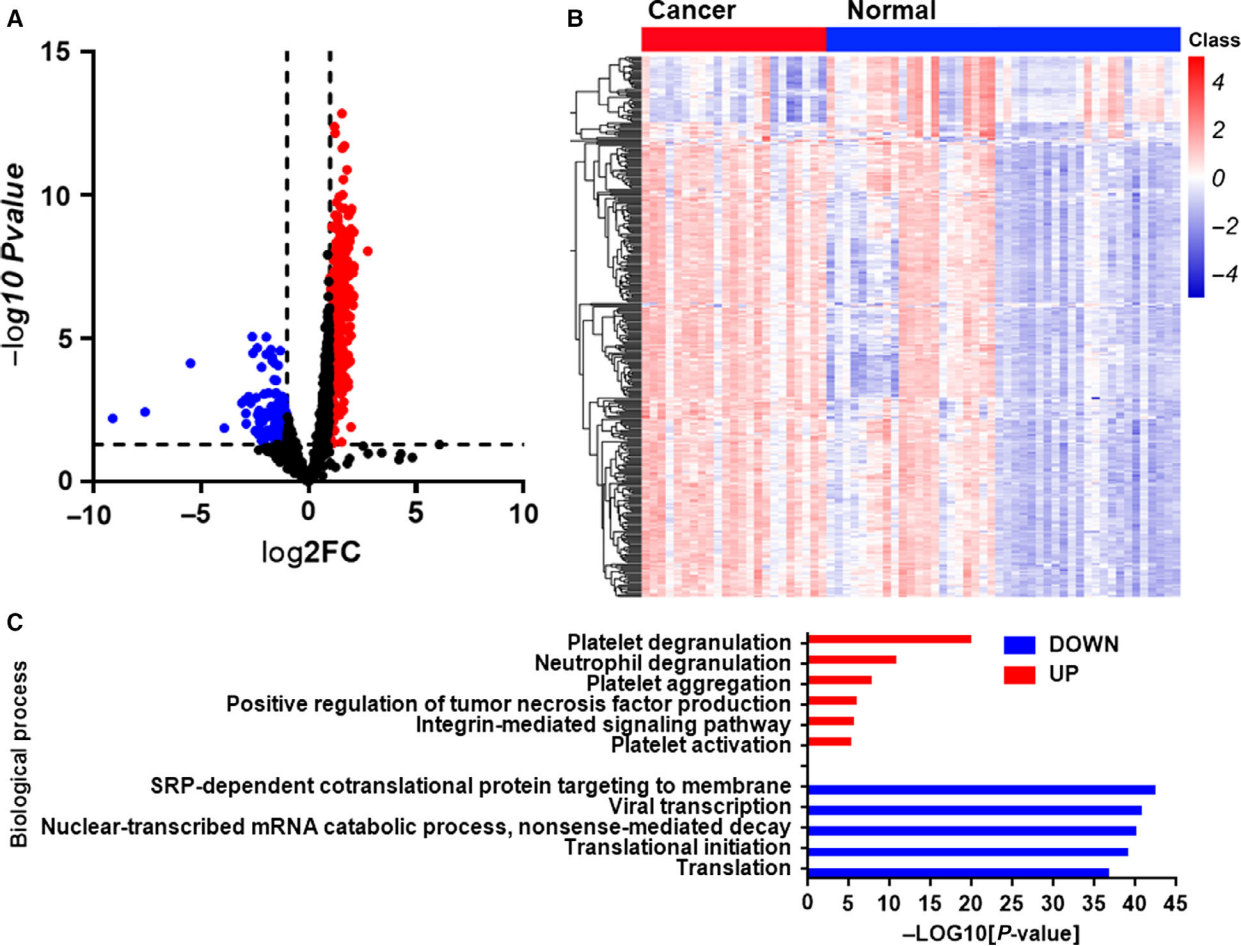
Identification of mRNAs in platelets from patients with BC and normal control subjects in the screening phase. (A, B) Volcano plots (A) and heatmap (B) of mRNAs in platelets from patients with BC and normal controls. (C) Gene Ontology enrichment analysis of up‐regulated and down‐regulated genes. FC, fold change; SRP, signal recognition particle.

To investigate the potential diagnostic biomarkers from platelet RNA profiles for BC, we performed high‐throughput sequencing with Illumina HiSeq 2500 of platelet samples from 23 patients with BC and 45 healthy volunteers. We identified a total of 138 up‐regulated gene mRNAs and 18 down‐regulated gene mRNAs between patients with BC and healthy volunteers with average reads > 1000, *P* < 0.05 and fold change > 2. The volcano plot and heatmap showed that the RNA profiles in platelets could make an obvious distinction between patients with BC and healthy volunteers (Fig. 1A,B). To further assess the differentially expressed genes in the platelets, we therefore aimed to explore functional variation between the two groups using funrich software 13. As shown in Fig. 1C, most up‐regulated genes are involved in platelet degranulation, aggregation and activation, which were confirmed to be able to promote tumor progression, whereas the down‐regulated genes are related to translation and transcription.

### Integrative network analysis reveals the hub genes

**Figure 2:**
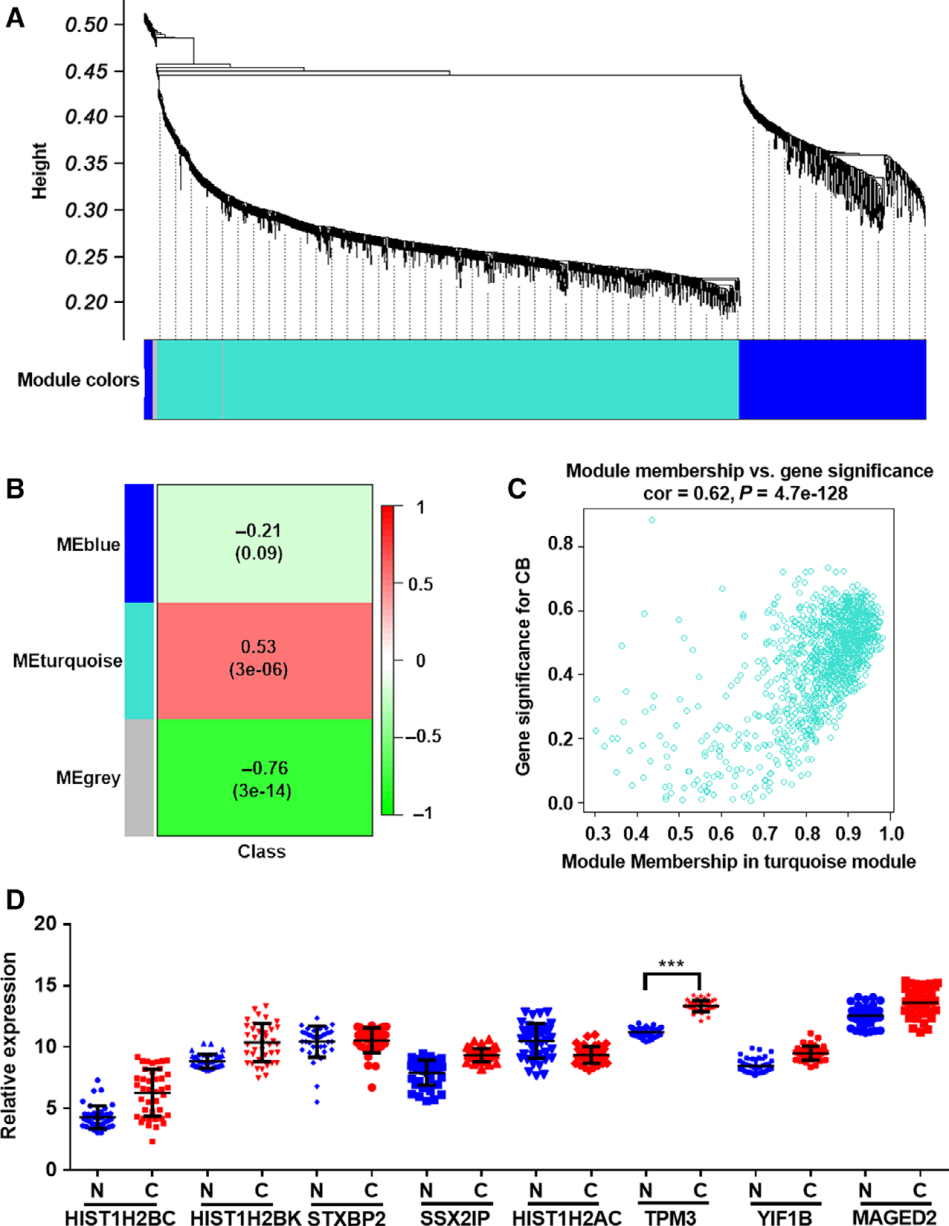
WGCNA screening of hub genes of platelet RNAs from patients with BC and healthy subjects. (A) Clustering dendrograms of genes, with dissimilarity based on topological overlap, together with assigned module colors. (B) Module‐trait associations. Each row corresponds to a module eigengene, and each column corresponds to a trait. Each cell contains the corresponding correlation and *P*‐value. The table is color coded by correlation according to the color legend. (C) A scatterplot of gene significance (GS) for subtype versus module membership (MM) in the turquoise module. (D) The relative levels of eight hub genes in platelets from 40 normal controls (*n* = 40) and 40 patients with BC (*n* = 40) by qRT‐PCR. Each point represents the mean of triplicate samples. Each *P*‐value was calculated with a nonparametric Mann–Whitney test: ***P < 0.001. CB, cancer biomarker.

To further investigate whether these hub genes in the platelets were potential biomarkers for BC diagnosis, we performed the WGCNA of the differentially expressed genes in the platelets from patients with BC and healthy subjects. Our findings uncover a total of three functional modules (blue, turquoise and gray modules) (Fig. 2A). The gray module would not be analyzed further because it had no association with the disease as previously reported. As shown in the matrix of Module‐Trait Relationships (Fig. 2B,C), the turquoise module showed a positive association (*r* = 0.53; *P* = 3E−6) with BC. Using the gene significance and module membership measures, the turquoise module also showed a significantly higher difference between patients with BC and healthy subjects (*r* = 0.62; *P* = 4.7E−128). Our findings uncover that the turquoise modules may be a key platelet‐derived mRNA module in BC. Then, the signedKME and module Eigengenes functions analysis in WGCNA package were applied to determine the hub mRNAs in the turquoise module. As shown in Table 3, a total of eight hub genes (*HIST1H2BC*, *HIST1H2BK*, *STXBP2*, *SSX2IP*, *HIST1H2AC*, *TPM2*, *YIF1B* and *MAGED2*) were investigated from the turquoise modules. These genes in the platelets may be functionally involved in the control of BC progression and serve as potential biomarkers for BC diagnosis.

**Table 3:** The primers used for qRT‐PCR.

Gene	Forward (3′–5′)	Reverse (3′–5′)
*HIST1H2BC*	ACCTCCAGGGAGATCCAGAC	AGCTGGTGTACTTGGTGACG
*HIST1H2BK*	AACAAGCGCTCGACCATCA	CCTTTGGGGTTGGGCTTTA
*STXBP2*	ATTCTGAGCGGAGTTATTCGGA	CCGCCGTTTGTTGATGTCTTC
*SSX2IP*	CCGGGGAACTAAGCAGAGAGA	GTTCATGGTCTTGTCGTGAGAT
*HIST1H2AC*	GCGACAACAAGAAGACTCGC	CGTTTCCGGGAGCTCAGATA
*TPM3*	TGAAAACCGGGCCTTAAAAGAT	GATCACCAACTTACGAGCCAC
*YIF1B*	GCTGTGGACACCATGTATGTG	CAGCCACCAAAACGTAGGTGA
*MAGED2*	ACAAAGGTCAATACAAAGGCTCA	GGGCCGAGTATCCTGATTCTC

### Validation of different expressed mRNAs in platelets via RT‐PCR assay

**Figure 3:**
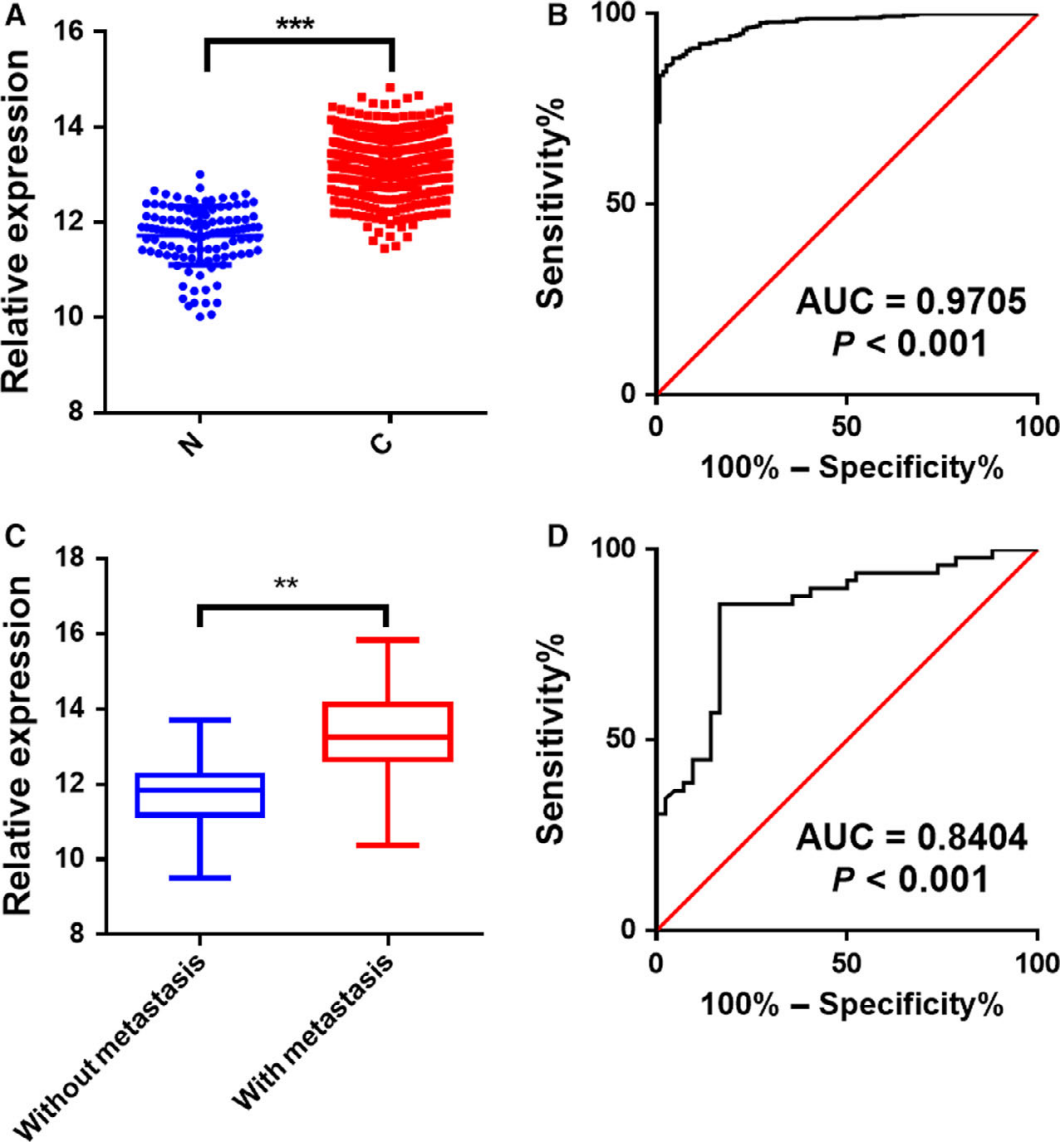
The relative levels and ROC analysis of *TPM3* mRNAs in the platelets. (A, B) The relative levels (A) and ROC analysis (B) of *TPM3* mRNAs in the platelets from normal individuals (*n* = 109) and patients with BC (*n* = 504) by qRT‐PCR. (C, D) The relative levels (C) and ROC analysis (D) of *TPM3* mRNAs in the platelets in patients with BC with (*n* = 49) or without (*n* = 42) metastasis. Each point represents the mean of triplicate samples. Each *P*‐value was calculated with a nonparametric Mann–Whitney test: **P < 0.01, ***P < 0.001.

Next, to investigate whether these eight candidate mRNAs could be used as biomarkers for BC diagnosis, we utilized qRT‐PCR assays to evaluate the WGCNA in two independent cohorts. We first measured the concentrations of the eight candidate mRNAs in the platelets from 45 patients with BC and 45 healthy subjects. As shown in Fig. 2D, the expression levels of *TPM3* mRNA were significantly increased in the platelets from patients with BC with the mean >2.0‐fold increase and the *P*‐value < 0.05, whereas the other seven mRNAs showed no difference between patients with BC and healthy subjects. Further investigation is needed to better understand the expression patterns of *TPM3* in a larger cohort (validation set), including 504 patients with BC and 109 healthy subjects. In accordance with the results of the training set, the concentrations of *TPM3* mRNA in the platelets were also significantly increased in patients with BC compared with 109 healthy subjects (Fig. 3A). Subsequently, we applied ROC curve analysis to investigate the diagnostic value of the *TPM3* mRNA in the platelets for BC (Fig. 3B). The area under the curve of *TPM3* mRNA was 0.9705 (95% CI: 0.9494–0.9823) (Fig. 3B). Our findings provide compelling evidence that *TPM3* mRNA in the platelets exerts a relatively high diagnostic accuracy for BC.

### *TPM3* mRNAs in the platelets serve as metastatic or local BC biomarkers

It is well established that metastasis is the major leading cause of cancer‐related death, including BC. Therefore, we investigated whether the *TPM3* mRNAs in the platelets could serve as biomarkers for the diagnosis of metastatic BC. As shown in Fig. 3C, expression of *TPM3* mRNAs was significantly higher in the platelets from patients with metastasis than in the metastasis‐free patients (*P* < 0.001). The area under the curve was 0.8404 (95% CI: 0.7566–0.9242) (Fig. 3D). These results demonstrate that the *TPM3* mRNAs in the platelets are closely associated with BC metastasis and may be exploited as auxiliary indicators for the metastatic BC.

### Tumor‐educated platelets promote BC cell invasion by delivering *TPM3* mRNA into cancer cells through microvesicles

**Figure 4:**
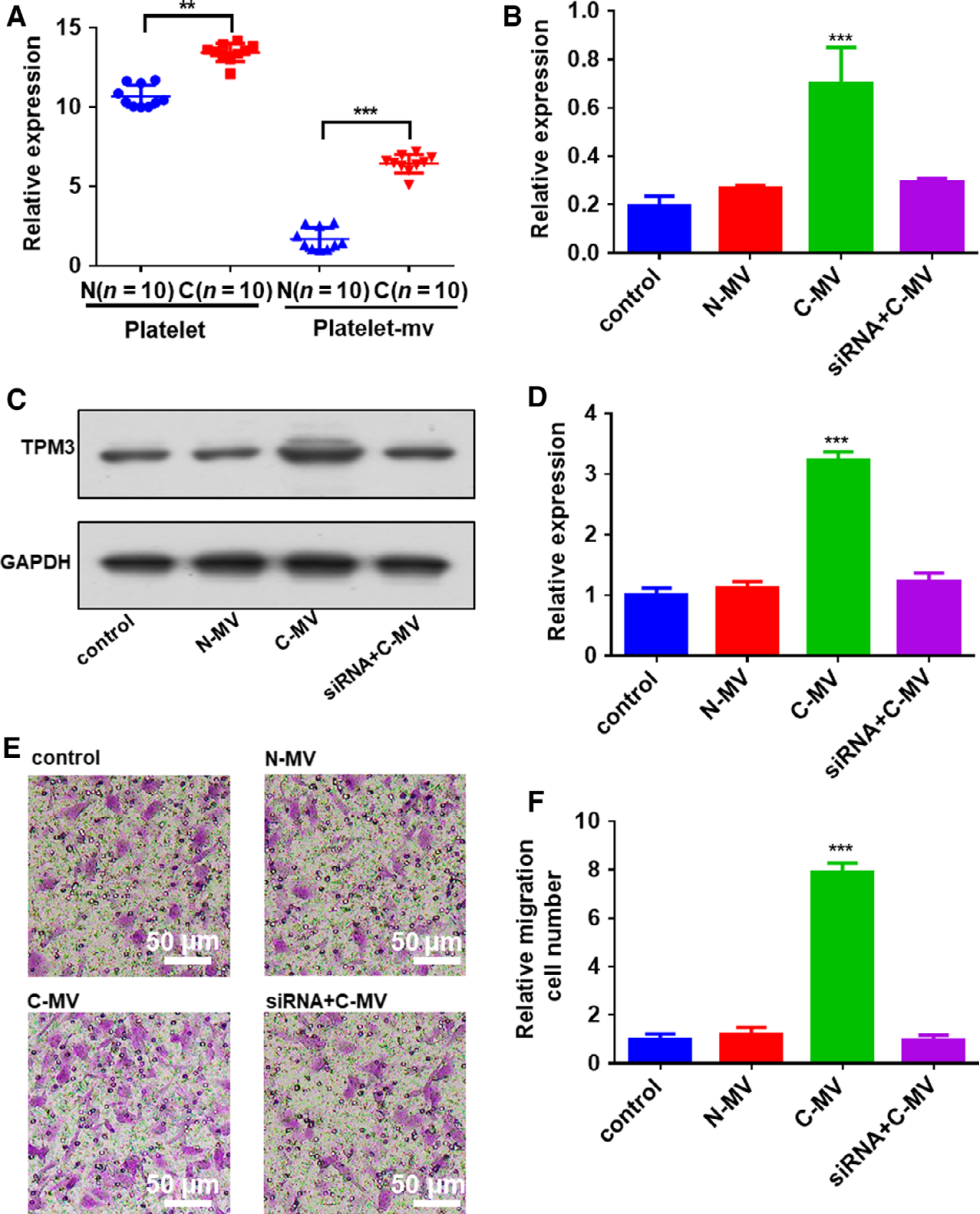
Platelet microvesicles from patients with cancer promote cancer cell migration by delivering *TPM3* mRNA. (A) The mRNA level of *TPM3* in platelets and platelet microvesicles (platelet‐mv) from 10 normal individuals and 10 patients with BC. (B) The mRNA level of *TPM3* in MDA231 cells exposed to platelet microvesicles from healthy volunteers and patients with cancer. (C, D) The protein level of *TPM3* in MDA231 cells exposed to platelet microvesicles from healthy volunteers and patients with cancer. (C) Representative image. (D) Quantitative analysis. (E, F) Platelet microvesicles from patients with cancer promote cancer cell migration. (E) Representative image (scale bars: 50 μm). (F) Quantitative analysis. Data are shown as the means ± standard error of the mean (*n* = 3). Statistical analysis was performed by two‐tailed Student’s *t*‐test; **P < 0.01, ***P < 0.001.

Previous studies suggested that microvesicles were secreted by activated eukaryotic cells, including the platelets and red blood cells without nucleus. Recent studies provide strong evidence that microvesicles derived from platelets play pivotal roles in the crosstalk between platelets and cancer cells by delivering RNAs and proteins 14. We compared the *TPM3* mRNAs in the platelets and platelet‐releasing microvesicles from 10 healthy subjects and patients with BC. In line with previous studies, we found that *TPM3* mRNAs were significantly elevated both in platelets and in platelet‐releasing microvesicles in the patients with BC (Fig. 4A). More interestingly, the fold change of platelet‐releasing microvesicles is much bigger than the fold change of platelets, because the *TPM3* mRNAs appeared to be very low level in the platelet‐releasing microvesicles from the healthy subjects (Fig. 4A). Moreover, the TPM3 proteins in the platelet‐releasing microvesicles were also investigated by western blotting. The results showed there were almost no TPM3 proteins in the platelet‐releasing microvesicles from patients with BC or in the platelet‐releasing microvesicles from the healthy subjects (Fig. S1). Subsequently, we collected platelet‐releasing microvesicles from patients with BC and healthy subjects, and incubated the microvesicles with cultured MDA231 cells. As shown in Fig. 4B, *TPM3* mRNA was significantly up‐regulated when MDA231 cells were incubated with the platelet‐releasing microvesicles from patients with BC. In contrast, the levels of the *TPM3* pre‐mRNA in the recipient cells were not altered (Fig. S2), implying that the effects of microvesicles on the expression of *TPM3* mRNA were caused by the platelet‐releasing microvesicles delivery and not because of *de novo* transcription. Moreover, the elevation could be abolished by cotransfecting MDA231 cells with *TPM3* small interfering RNAs (siRNAs) (Fig. 4B). The efficiency of *TPM3* siRNAs was shown in Fig. S3A–E. According to our observations, we then aimed to determine whether the invasion of MDA231 cells was enhanced by transferring the *TPM3* mRNAs into cancer cells through platelet‐releasing microvesicles. Western blotting was performed to analyze the protein level of TPM3 in the MDA231 cells incubated with platelet‐releasing microvesicles from healthy subjects or patients with BC. The results showed incubation of MDA231 cells with platelet‐releasing microvesicles from patients with BC significantly increased the levels of cellular TPM3 protein, and this up‐regulation was dramatically abolished by cotransfection with *TPM3* siRNAs (Fig. 4C,D).

*TPM3*, a member of the tropomyosin family, encodes an actin‐binding protein. A number of observations suggested that *TPM3* contributes to tumor metastasis 15, 16, 17. Consistent with previous studies, we found the exogenous *TPM3* delivered by platelet‐releasing microvesicles could enhance the tumor cell invasion *in vitro* by transwell assay. As in the previous study 15, 16, MDA231 cells transfected with *TPM3* siRNA showed a decreased invasion (Fig. S3D,E). Given the role of *TPM3* in promoting the invasion of tumor cells, we examined whether the effect of platelet‐releasing microvesicles from patients with BC on the invasion of MDA231 cells was *TPM3* dependent. As expected, the invasive ability of MDA231 cells was sharply enhanced because of the platelet‐releasing microvesicles from patients with BC (Fig. 4E,F), whereas the up‐regulation of invasive activity was attenuated by silencing *TPM3* with siRNAs (Fig. 4E,F).

## Discussion

BC is the second most commonly diagnosed cancer in women worldwide, with an increasing incidence in Third World countries 1. Despite the recent advances in both diagnosis and treatment, in most patients, BC eventually develops into distant metastasis in advanced cancers. Because metastasis is the principle cause of cancer mortality, novel noninvasive biomarkers are urgently needed for early diagnosis of BC.

The involvement of platelets in tumor growth and metastasis is becoming a research hotspot in the field of oncology biology 2. A number of observations suggest that platelets can be educated by cancer cells [named tumor‐educated platelets (TEPs)] and modulate the content of their RNAs or absorb RNAs of tumors in response to signals from cancer cells, resulting in changes in the transcriptome profiles that could reflect pathological progressions 18. These TEP RNAs have been emerging as potential novel biomarkers for cancer diagnosis, prognosis and prediction 18. In 2010, Calverley *et al*. 7 employed microarray analysis to profile the mRNA in the platelets from healthy individuals and patients with lung cancer. They found a total of 197 genes (99%) were significantly decreased in platelets of patients with lung cancer, and 608 splicing events showed differences between the metastasis and negative control groups 7. Subsequently, Nilsson *et al*. 8 demonstrated that tumor cells could transfer RNAs into platelets, and confirmed platelets isolated from patients with glioma and prostate cancer contain the cancer‐associated RNA biomarkers EGFRvIII and PCA3, respectively. In addition, they further revealed by gene expression profiling a distinct RNA signature in platelets from patients with glioma compared with normal control subjects 8. In a follow‐up study, they found that platelets can sequester EML4–ALK rearrangements from tumor cells and be detectable by qRT‐PCR 9. More interestingly, the detection and persistence of EML4–ALK rearrangement in platelets were closely associated with shorter progression‐free survival to crizotinib 9. In 2015, Best *et al*. 10 performed RNA sequencing of 55 healthy controls and 228 patients with different tumor types, including BC, and developed a machine learning‐based classification algorithm allowing for pan‐cancer diagnostics with an accuracy rate of > 95% and tumor type pinpointing with an accuracy rate of > 70% base on the platelet RNA profiles. Moreover, the spliced RNA surrogate signatures of platelets were also found to be associated with the tumor tissue molecular subtype, such as EGFR and KRAS mutations in lung cancer and HER2 and MET amplifications in BC with 85–95% accuracy rates, respectively. This proof‐of‐concept study was followed by a follow‐up study that included additional analysis of age‐matched cohorts and patients with NSCLC 11. In this follow‐up study, they found that the particle‐swarm optimization‐enhanced algorithms enable efficient use for early‐ and late‐stage NSCLC diagnoses based on the platelet RNA profile 11.

In this study, we characterized the gene expression signatures of platelets in BC by high‐throughput sequencing and qRT‐PCR assays. Our results suggested that the expression of *TPM3* mRNA was significantly elevated in the platelets from patients with BC, compared with the age‐matched healthy controls. Besides detection of cancer, RNAs of TEPs were also found to be used for the monitoring of cancer progression because platelets could sequester tumor‐derived RNA molecules. Nilsson *et al*. 9 found that the EML4–ALK fusion transcripts in the platelets are much lower in the patients with NSCLC with the effective anti‐EML4–ALK crizotinib therapy compared with the patients without response for the anti‐EML4–ALK crizotinib therapy. Notably, we found that *TPM3* mRNA in the platelets was delivered into BC cells through microvesicles and led to enhanced migrative phenotype of BC cells. Due to the lifespan of a regular platelet of ~ 7–10 days, TEP RNA analysis might reveal an up‐to‐date, enhanced and dynamic reflection of the activity of BC, such as metastasis, therapeutic effect. However, these findings warrant further investigation in studies with a larger sample size.

A number of studies suggest that platelet‐derived microvesicles have a surface with 50‐ to 100‐fold higher specific procoagulant than activated platelets, and could transport and deliver bioactive molecules (such as RNAs and proteins) into tumor cells, thus participating in tumorigenesis and cancer progression 19. Previous studies suggest that platelet‐derived microvesicles could control the adhesion, survival and proliferation of tumor cells by transferring their membrane receptors, such as cytokine receptors and platelet–endothelium adhesion receptors 20. In addition to proteins, platelet‐derived microvesicles can also deliver miRNAs into tumor cells and modulate multiple inflammatory responses and signaling pathways 21. For example, miR‐223, transferred by platelet‐derived microvesicles into lung cancer cells, has been proved to induce the invasion of A549 cells by suppressing EBP41L3 22. Tang *et al.* 23 found that platelet‐derived microvesicles could transfer miR‐939 to ovarian epithelial cancer cells and contribute to the epithelial‐to‐mesenchymal transition (EMT) of SKOV3 cells, which is involved in invasion and metastasis of tumors. Similar to previously published studies, Michael *et al*. 24 revealed platelet‐derived microvesicles could infiltrate into solid tumors and deliver miR‐24 to tumor cells, inducing cell apoptosis and suppressing tumor growth by down‐regulating mt‐Nd2 and Snora75. In this study, we first reported platelet‐derived microvesicles could also transfer *TPM3* mRNA into BC cells. *TPM3*, as a member of the tropomyosin family of actin‐binding proteins, is involved in the contractile system of striated and smooth muscles and the cytoskeleton of nonmuscle cells. Mutations in this gene result in autosomal dominant nemaline myopathy, and oncogenes formed by chromosomal translocations involving this locus are associated with multiple cancers, including BC. Multiple studies have proved that *TPM3* could promote the invasion of tumor cells 17, 25, 26, 27, 28. Consistent with previous studies, our results showed the expression level of *TPM3* mRNA in the platelets was significantly increased in the platelets from patients with metastasis compared with the patients without metastasis. By transwell assay, we also revealed that the *TPM3* mRNA delivered by platelet‐derived microvesicles could be translated into proteins in the cancer cells and promote invasion. Detection of metastasis is critical for patients with BC, because patients with metastatic BC often predict a poorer overall survival than that of patients without metastasis. We next characterized the expression patterns of *TPM3* mRNAs in the platelets through TEP RNA‐sequencing analysis and qRT‐PCR assays. Multiple lines of evidence of this study indicated that *TPM3* mRNA was significantly increased in patients with metastatic cancer and could be delivered into cancer cells by microvesicles to promote the invasion of BC cells.

Our findings revealed the relationships between *TPM3* mRNA in the platelets and the metastasis of BC cells, and the *TPM3* mRNA in the platelets can be exploited as biomarkers for early diagnosis of metastatic BC, thereby extending the life expectancy of patients with BC.

## Conflict of interest

The authors declare no conflict of interest.

## Author contributions

QZ designed the experiments. BY, SQ, RH, WG and SJ performed the experiments and analyzed the results. JJ wrote the manuscript.

## Supporting information


**Fig. S1.** The protein level of TPM3 in the MDA231 cells, N‐MVs (microvesicles released by platelets derived from healthy subjects) and C‐MVs (microvesicles released by platelets derived from patients with BC) by western blotting.
**Fig. S2.** The level of *TPM3* pre‐mRNA in the MDA231 cells incubated with or without platelet‐releasing microvesicles. Data are shown as the means ± standard error of the mean (*n* = 3). Statistical analysis was performed by two‐tailed Student’s *t*‐test.
**Fig. S3.** The efficiencies of siRNA of *TPM3*. (A) The mRNA level of *TPM3* in MDA231 cells transfected with scramble RNA or siRNA. (B, C) The protein level of TPM3 in MDA231 cells transfected with scramble RNA or siRNA. (B) Representative image. (C) Quantitative analysis. (D, E) The transwell assay of MDA231 cells transfected with scramble RNA or siRNA. (D) Representative image (scale bars: 50 μm). (E) Quantitative analysis. Data are shown as the means ± standard error of the mean (*n* = 3). Statistical analysis was performed by two‐tailed Student’s *t*‐test; *P < 0.05, **P < 0.01.

## Data Availability

All data generated or analyzed during this study are included in this article. Raw and processed data are stored in the laboratory of globaldata bank and are available upon request.
