# Supplementary material for: Delivery of platelet TPM3 mRNA into breast cancer cells via microvesicles enhances metastasis
Source: FEBS Open Bio. 2019 Nov 21;9(12):2159–69. doi: 10.1002/2211-5463.12759 (PMC6886296; doi:10.1002/2211-5463.12759)
Supplement: Supplementary file 1 — Fig. S1. The protein level of TPM3 in the MDA231 cells, N‐MVs (microvesicles released by platelets derived from healthy subjects) and C‐MVs (microvesicles released by platelets derived from patients with BC) by western blotting. Fig. S2. The level of TPM3 pre‐mRNA in the MDA231 cells incubated with or without platelet‐releasing microvesicles. Data are shown as the means ± standard error of the mean (n = 3). Statistical analysis was performed by two‐tailed Student’s t‐test. Fig. S3. The efficiencies of siRNA of TPM3. (A) The mRNA level of TPM3 in MDA231 cells transfected with scramble RNA or siRNA. (B, C) The protein level of TPM3 in MDA231 cells transfected with scramble RNA or siRNA. (B) Representative image. (C) Quantitative analysis. (D, E) The transwell assay of MDA231 cells transfected with scramble RNA or siRNA. (D) Representative image (scale bars: 50 μm). (E) Quantitative analysis. Data are shown as the means ± standard error of the mean (n = 3). Statistical analysis was performed by two‐tailed Student’s t‐test; *P < 0.05, **P < 0.01. [file FEB4-9-2159-s001.doc]

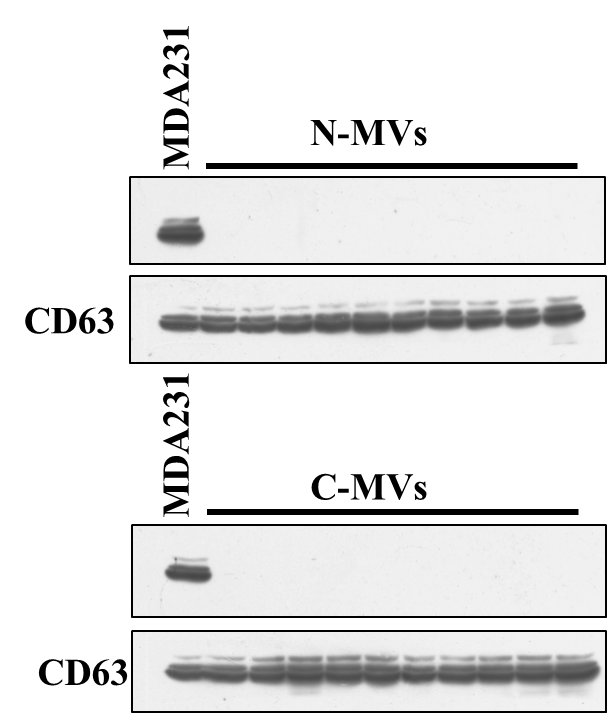


Figure s1. The protein level of TPM3 in the MDA231 cells, N-MVs (microvesicles released by platelets derived from healthy subjects), and C-MVs (microvesicles released by platelets derived from breast cancer patients) by western blotting.


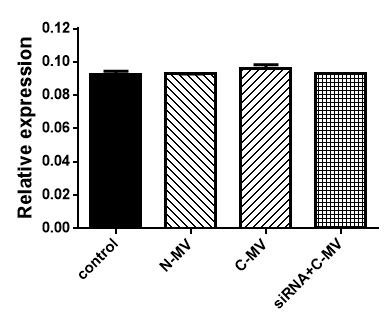


Figure s2. The level of TPM3 pre-mRNA in the MDA231 cells incubated with or without platelet-releasing microvesicles. Data are shown as the means ± standard error of the mean (n = 3), statistical analysis was performed by two-tailed Student’s t-test.


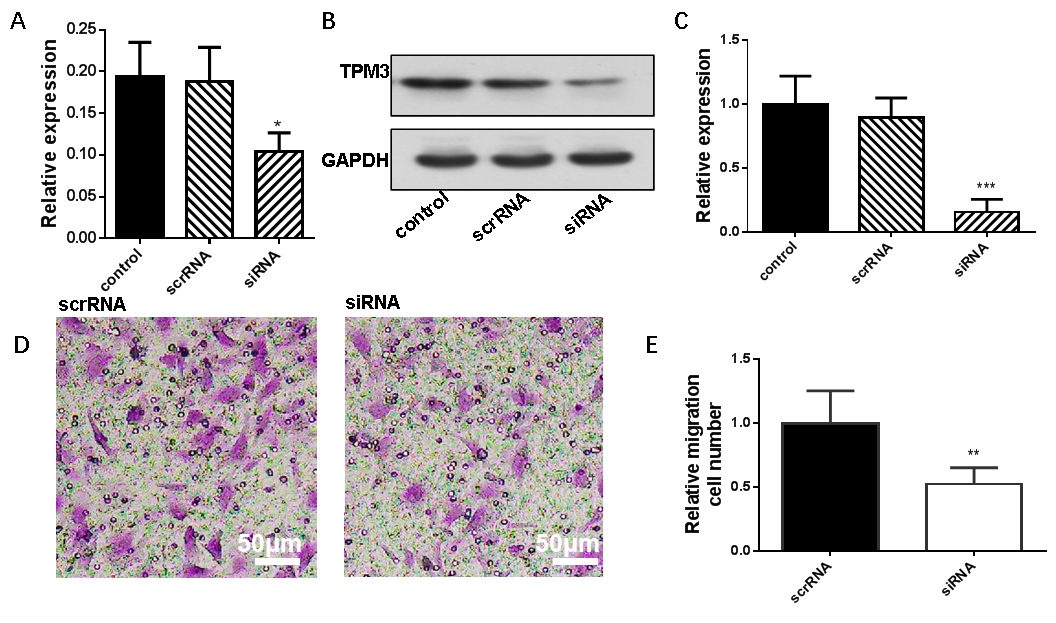


Figure s3. The efficiencies of siRNA of TPM3. (A) The mRNA level of TPM3 in MDA231 cells transfected with scramble RNA or siRNA. (B-C) The protein level of TPM3 in MDA231 cells transfected with scramble RNA or siRNA. B: representative image; C: quantitative analysis. (D-E) The transwell assay of MDA231 cells transfected with scramble RNA or siRNA. D: representative image （scale bars: 50μm）; E: quantitative analysis. Data are shown as the means ± standard error of the mean (n = 3), statistical analysis was performed by two-tailed Student’s t-test; *P < 0.05, **P < 0.01.
